# Challenges and Current Trends in Preventing Antimicrobial Resistance in EU Water Law Context

**DOI:** 10.3390/antibiotics14010018

**Published:** 2024-12-31

**Authors:** Justyna Rogowska, Grażyna Gałęzowska, Agnieszka Zimmermann

**Affiliations:** 1 Division of Pharmaceutical and Medical Law, Department of Social Medicine, Faculty of Health Sciences, Medical University of Gdansk, Tuwima Str. 15, 80-210 Gdansk, Poland; agnieszka.zimmermann@gumed.edu.pl; 2 Division of Bioenergetics and Physiology of Exercise, Faculty of Health Sciences, Medical University of Gdansk, Debinki 1 Str., 80-211 Gdansk, Poland; grazyna.galezowska@gumed.edu.pl; 3 Department of Environmental Engineering Technology, Faculty of Civil and Environmental Engineering, Gdansk University of Technology, Narutowicza Str. 11/12, 80-233 Gdansk, Poland

**Keywords:** antimicrobial resistance, water law, environment, European Union

## Abstract

The increasing consumption of pharmaceuticals, including antibiotics, and their improper disposal have resulted in both pharmaceuticals and their metabolites being released into the environment, where they pose a risk to both ecosystems and human health. One of the most serious threats to public health associated with the presence of antibiotics in the environment is antimicrobial resistance (AMR). In order to combat AMR, the legal aspect of water protection becomes a critical area of action. This article analyzes the current challenges and legislative developments in the European Union (EU) aimed at mitigating pharmaceutical contamination in aquatic environments, particularly with regard to AMR. It traces the evolution of EU water protection policies from the initial surface and groundwater directives to the recent updates of the Water Framework Directive, Groundwater Directive and Environmental Quality Standards Directive, focusing on the integration of pharmaceutical contaminants into the regulatory framework. In addition, these changes include the update of the Watch List system for monitoring emerging contaminants, the adoption of effects-based methods (EBMs) in the assessment of water status and the streamlining of the legislative process to respond more quickly to emerging threats in the aquatic environment. The EU’s strategic approach to pharmaceuticals in the environment is emphasized as a key framework for harmonizing the environmental standards and addressing the problem of AMR through more sustainable pharmaceutical practices. This study advocates for a proactive, integrated approach to water policy that aligns regulatory actions with scientific advancements to protect public health and ecosystem integrity.

## 1. Introduction

Pharmaceutical contaminants are biologically active compounds used in the treatment of humans and animals that enter the environment as a result of both production, use and improper disposal. These compounds are one of the classes of micropollutants, defined as contaminants detected in the environment in low concentrations [1]. The presence of pharmaceuticals in the environment has been a topic of increasing concern for many years [2,3,4,5]. The growing demand for pharmaceuticals is linked to population growth, longer life expectancy and economic development, all of which are associated with an increase in chronic diseases [6]. According to the World Health Organization (WHO), global life expectancy increased from 66.8 years in 2000 to 71.4 in 2021 and is projected to reach 78.2 by 2050 [7]. At the same time, the leading causes of death worldwide—cardiovascular diseases, respiratory infections and tuberculosis—are expected to persist through 2050 [8]. The growing pharmaceutical demand has led to significant environmental challenges, particularly in aquatic ecosystems. Pharmaceuticals enter the environment through human excretion (metabolites) or improper disposal [9]. Additionally, they can enter water systems from livestock farms, aquaculture, pharmaceutical manufacturing and stormwater runoff [10]. Due to the fact that conventional wastewater treatment is not able to completely remove pharmaceuticals from wastewater, they end up in water reservoirs (rivers and lakes), which are often a source of drinking water for residents. Among pharmaceuticals, we distinguish numerous categories such as analgesics, antidepressants, beta-blockers, diuretics, hormones or antimicrobials (antibiotics, antiviral and antifungal agents) [11]. One of these categories is antibiotics, which are used in both human and veterinary medicine [11,12]. One of the most pressing public health risks associated with the excessive use and improper disposal of antibiotics is antimicrobial resistance (AMR), which renders antibiotics less effective [13,14]. AMR is a phenomenon in which microorganisms such as bacteria become increasingly resistant to antimicrobial agents to which they were previously susceptible [14]. According to the WHO, AMR is one of the greatest global threats to public health and, in 2019, was directly responsible for 1.27 million deaths worldwide and contributed to 4.95 million deaths [13]. The European Centre for Disease Prevention and Control indicates that approximately 33,000 patients die each year from antibiotic-resistant bacteria in the European Union and in the European Economic Area. Estimates indicate that AMR could cause more deaths worldwide than cancer by 2050 [13].

The presence of antibiotics in the environment has a significant impact on the development of AMR. Therefore, limiting the release of pharmaceutical contaminants into the environment, especially the aquatic environment, is a key element of the strategy to prevent/reduce the AMR problem. In response to growing concerns about AMR and its connection with environmental pollution with pharmaceuticals, the EU has begun to introduce changes in the legal regulations regarding the monitoring of water quality and pollution management. Given the global importance of the AMR problem, especially in terms of sustainable development and public health, the legal aspect of water protection becomes a critical area of action. Therefore, this article aims to analyze the current EU legislation on the protection of water from pharmaceutical contamination in the context of preventing AMR. In addition, the evolution of AMR strategies and regulations regarding the protection of water from pharmaceutical contamination are presented. Problems and challenges that may arise during the implementation of new legislative solutions are also discussed.

## 2. AMR: Past and Present

In order to counteract AMR in 1998, the World Health Assembly (WHA), in its resolution, called for Member States to rationally use antimicrobials in both human and veterinary medicine, to increase supervision over the sale of antimicrobials and to intensify actions to counteract the spread of infections [15]. The first global WHO plan to combat AMR was published in 2001 as the *Global Strategy for Containment of Antimicrobial Resistance*, which included 68 recommendations aimed at actions aimed at patients, physicians, healthcare facilities and national authorities [16]. Many of these actions were also included in the *Global Action Plan on AMR* adopted by the WHO in 2015. The plan required countries to develop and implement multi-sectoral national action plans to combat antimicrobial resistance by reducing the incidence of infections, optimizing the use of antimicrobials and increasing education about the problem of AMR in the health, veterinary and agricultural sectors [16].

In parallel, actions were taken in the EU to combat drug resistance. Although, according to Article 168 Treaty on the Functioning of the European Union (TFEU), public health protection is the responsibility of the Member States, EU actions are to complement and support national policies [17]. Already in 2001, the European Commission issued *Communication from the Commission on a Community Strategy* against antimicrobial resistance, in which it points out the need to introduce a community strategy concerning four key areas of action, including a surveillance system for antimicrobial resistance and the use of antimicrobials in both human and veterinary medicine, and reducing the need for antimicrobials by preventing the spread of infectious diseases and controlling infections [18]. As a result, EU authorities have taken regulatory action to prevent drug resistance, including a ban on the use of antibiotics other than coccidiostats and histomonostats to stimulate growth in feed since 1 January 2006 [19]. In 2011, the European Commission adopted the first *Action Plan Against the Rising Threats from Antimicrobial Resistance*, containing 12 actions [20]. Although the plan was based on a holistic approach to combat and limit the spread of AMR, it did not directly address the role of the environment in this regard but focused more on limiting the use of drugs in human and veterinary medicine or the need to develop new, more effective antimicrobial drugs. However, the European Commission indicated the need for international cooperation in reducing environmental pollution with drugs, primarily from manufacturing plants [20]. The emphasis on the role of the environment in the development and spread of AMR was reflected in *A European One Health Action Plan Against Antimicrobial Resistance*, which was adopted by the European Commission in June 2017. The plan indicated the need to conduct research, including monitoring, on AMR related to the environment and to adopt a strategy on pharmaceuticals in the environment, including the aquatic environment [21]. The plan also highlights the importance of the Scientific Committee on Health and Environmental Risks (SCHER), established in March 2004 [22], as a body responsible for providing knowledge on AMR related to the environment [21]. The clear emphasis that human and animal health and the environment are interconnected and that the environment can be another source of drug-resistant microorganisms was made in the resolution of 13 September 2018 on *A European One Health Action Plan Against Antimicrobial Resistance* [23]. In its resolution, the European Parliament called on the European Commission to immediately develop an EU strategy to combat pharmaceutical residues in water, which will focus on monitoring pharmaceuticals in the environment, collecting monitoring data or analyzing the effects of AMR on water resources. Therefore, protecting water from pollution caused by pharmaceuticals, including antibiotics, is one of the elements of the strategy to counter AMR.

## 3. Water Policy: Past, Present and Future

### 3.1. Surface Water—Past and Present

The first EU directives on the protection of water against pollution appeared in 1975 and 1976. The first one concerned the quality of surface waters used as a source of drinking water, while the second one concerned the introduction of hazardous substances into surface water [24,25,26]. In order to further ensure water protection, in 1991, additional directives concerning pollution from urban wastewater and nitrates from agricultural runoff were introduced to further safeguard water quality [27]. The first directive establishing a framework for the protection of all types of water, except for marine waters, i.e., groundwater, inland surface waters, estuarine waters and coastal waters, is Directive 2000/60/EC (commonly known as the Water Framework Directive; WFD) [28]. Marine waters were later incorporated into the EU legal system through Directive 2008/56/EC (Marine Strategy Framework Directive; MSFD) [29]. The aim of the WFD was for Member States to achieve ‘good water status’ by reducing the emissions of priority substances, eliminating these substances from the aquatic environment and using water rationally in accordance with the principle of sustainable development. Therefore, a list of priority substances that may pose a particular risk to the aquatic environment has been developed. To determine whether a substance poses a significant risk to aquatic ecosystems, the following factors are taken into account:

- Scientific evidence that the substance may pose a risk (in particular, its ecotoxicity and human toxicity);
- Monitoring data that indicates the prevalence of a substance in the aquatic environment;
- Other reliable data such as the production levels of a substance, its use or application [28].

The first list of priority substances containing 33 compounds/groups of compounds was established in 2001 [30] and then updated in 2008 (Directive 2008/105/EC; EQSD) [31] and 2013 (Directive 2013/39/EU) [32]. Directive 2008/105/EC contained environmental quality standards (EQSs), which set limits on the concentrations of compounds identified as a priority in the policy field [31]. The EQSs were updated for 7 of the 33 compounds in Directive 2013/39/EU [32]. In addition, Directive 2013/39/EU extended the catalog of compounds by another 12. Member States are required to implement environmental quality standards for these 12 compounds by 22 December 2027 [32]. Pharmaceuticals, however, were not included on any of these lists. Despite the growing recognition of pharmaceuticals as environmental pollutants, the lack of sufficient data on their ecotoxicity and long-term effects delayed their inclusion on the priority substances list, underscoring the complexity of regulating these emerging contaminants. However, under Article 8b of Directive 2013/39/EU, the Watch List was established, which includes compounds that may pose a significant risk to the aquatic environment, but the lack of sufficient data or insufficient quality of data does not allow for their inclusion on the list of priority substances [32]. The first Watch List, published in 2015, included 17 compounds, including three macrolide antibiotics, three hormones and one non-steroidal anti-inflammatory drug (NSAID) [33] (Table 1). This list was updated in 2018 (amoxicillin and ciprofloxacin were added, and diclofenac was removed) and 2020 (sulfamethoxazole, trimethoprim, venlafaxine, o-desmethylvenlafaxine, and a group of 10 azole compounds were added, and 17-alpha-ethinylestradiol (EE2), 17-beta-estradiol (E2), estrone (E1), and macrolide antibiotics were removed) [34,35]. The latest Watch List was established in 2022 [36] (Table 1).

As already mentioned, the list of priority substances, the EQSs for these substances and the Watch List are subject to periodic review. The collection of data on the compounds on the Watch List is intended to show whether these compounds pose a risk to the aquatic environment and, therefore, should be included on the list of priority substances and whether EQSs should be established for them [37]. Compounds/groups of compounds are monitored for up to 4 years. During this period, EU countries are obliged to monitor the indicated substances at least once a year [38]. Then, based on the information collected, a decision is made to place them on the list of priority substances or remove them from the Watch List due to lack of information about the threat or insufficient monitoring data [39]. The Watch List is updated every 2 years. In accordance with article 16(4) of the WFD, the list of priority substances is updated at least every 4 years [28].

### 3.2. Groundwater—Past and Present

Groundwater plays a critical role in providing drinking water within the EU. It is estimated that 65% of drinking water in the EU is sourced from groundwater. Additionally, groundwater is essential for agricultural irrigation [40]. The first EU directive on groundwater was agreed in 1980 [41]. As Skinner [42] pointed out, this directive only influenced the waste management sector by raising the standards of water protection against pollution in landfills, but it had no significant impact on improving the management of groundwater. It was only the WFD that outlined a new framework for integrated protection measures for all types of water and became the starting point for the Directive on the Protection of Groundwater against Pollution and Deterioration (GWD), adopted in 2006 [43]. This directive specified more detailed measures for the protection of groundwater than those specified in the WFD, i.e., primarily the definition of groundwater quality standards and threshold values of pollutant concentrations affecting the assessment of the chemical status of groundwater. However, these standards did not refer in any way to pharmaceutical substances [43].

Published by the European Environment Agency (EEA) in 2018, a report on the state of water in Europe indicated that in 2015, only 38% of surface waters were in good chemical status, 46% did not achieve good status, and 16% of them had an unknown status. In the case of groundwaters, the analysis of data showed that 75% had good chemical status, 24% did not achieve good status, and in the case of 1%, this status was unknown. It is significant that since the previous assessment in 2009, the status of groundwater has not improved [44]. At the same time, in the same year, the European Parliament, in its Resolution of 13 September 2018 on the European One Health Action Plan against Antimicrobial Resistance, expressed concern that the European Commission had not presented a timely strategic approach to the presence of pharmaceuticals in water despite the obligation arising from the WFD. As a result, in 2019, the European Commission adopted the *Strategic Approach to Pharmaceuticals in the Environment* [45]. The legal obligation to develop a strategic approach to pharmaceuticals present in the environment resulted from Article 8c of Directive 2013/39/EU [32]. In addition, this strategy is one of the elements of the previously mentioned European *One Health Action Plan Against Antimicrobial Resistance (AMR)*. The main objective of the strategy is primarily to increase the level of knowledge both on the rational use of pharmaceuticals and the presence of pharmaceuticals in the environment and the risk resulting from this. In addition, the European Commission has committed to taking action to support the development of more ecological pharmaceuticals and their production methods. In the context of preventing microbial resistance, the Commission has indicated the need for further research on the relationship between the presence of pharmaceuticals in the environment and the development of antimicrobial resistance. At the same time, the Commission has determined that it will take pharmaceuticals and their groups more into account when reviewing both the list of priority substances and the Watch List [45]. Despite the initiatives taken by the European Commission and the Member States, the European Parliament considered that the actions taken so far are insufficient, as expressed in the European Parliament’s resolution of 17 September 2020 on the *Strategic Approach to Pharmaceuticals in the Environment* [46]. Although the Parliament considered that the strategic objectives presented by the European Commission in the *Strategic Approach to Pharmaceuticals in the Environment* were justified, it indicated that more effective actions were necessary to reduce the impact of pharmaceuticals on the environment. At the same time, the European Commission’s 2021 report on the implementation of the WFD indicated that only four Member States fully monitored the 12 substances that were added in 2013 and have identified the so-called main types of measures taken to reduce the impact of these substances on the environment. Another 11 countries monitored most of the priority substances. The Commission indicated that in the case of some Member States, the information submitted was incomplete, data for some substances were missing, substances were monitored in the wrong matrices, or there was no information on the sources of these substances [47].

### 3.3. Protection of Surface and Groundwater—The Future

Currently, water protection activities are part of both the European Green Deal, which is currently the main strategy in the field of environmental policy in the EU and whose goals include zero pollution by 2050, and the 2030 Agenda for Sustainable Development (especially goal 6–clean water and sanitation and 14–life below water) [48,49]. The action’s effect is to achieve a good groundwater chemical status by 2039. In the case of surface water, the deadline has not been extended, i.e., according to the WFD, Member States are obliged to achieve all environmental objectives by 2027.

#### 3.3.1. Legislative Updates and Strategic Changes

In October 2022, the European Commission published a proposal to amend three directives: the WFD, GWD and EQSD. On 24 April 2024, the European Parliament adopted its position in this case. The negotiation mandate was agreed by the Council of the European Union on 19 June 2024 and allows for a dialogue with the European Parliament on the final text of the amendments [50]. The purpose of these changes is to:

- Update the list of priority substances and quality standards for both groundwater and surface water;
- Place emphasis on monitoring not only individual compounds but also their mixtures, also in the context of seasonal differences in pollutant concentrations;
- Take action to make changes to legislation more quickly if scientific evidence appears indicating a risk resulting from the presence of a compound/group of compounds in the aquatic environment;
- Improve the consistency and transparency of data on pollutants and access to them [51].

Article 8c, added to the WFD, clearly indicates the serious threat to ecosystems resulting from the presence of pharmaceuticals in water. The European Commission’s *Strategic Approach to Pharmaceuticals in the Environment* (2019) emphasized the need for more comprehensive monitoring of pharmaceuticals and antimicrobial resistance genes [45]. This focus is evident in the updates to the Watch List and priority substances list, which are crucial to the broader goal of achieving better water quality under the European Green Deal.

#### 3.3.2. Monitoring Challenges and Data Collection

The European Parliament indicated that in order to include indicators of antimicrobial resistance evolution or transmission in the Watch List, not only reliable and harmonized monitoring methods must be developed, but also criteria for assessing these indicators must be developed based on scientific knowledge. At the same time, the European Parliament indicated that the use of these methods must not entail ‘excessive costs’ [51].

Table 2 below summarizes the key updates to the Watch List, highlighting the expanded scope and frequency of monitoring [51].

These Watch List updates are a critical part of improving data collection and ensuring more comprehensive monitoring of both surface and groundwater contaminants. Furthermore, updates to the priority substances list and environmental quality standards (EQS), which now include pharmaceuticals, represent a shift toward more stringent control measures. Table 3 summarizes the key updates to the priority substances list, including the establishment of national threshold values for groundwater contaminants [51].

The changes introduced in the directives are also intended to attempt to solve problems related to both the monitoring of priority substances and the collection of data on these substances. Data collection practices are inconsistent across EU Member States, with significant variations in the methodologies and frequencies of monitoring. While some countries, such as Germany and the Netherlands, have well-developed infrastructures for water quality monitoring and regularly update their systems to meet the EU directives, others struggle due to limited funding or technological capacity [52]. The EEA highlights that countries with a lower GDP (gross domestic product) tend to have weaker water quality monitoring frameworks, leading to significant disparities in data availability and reliability [53]. The Organization for Economic Cooperation and Development (OECD) and EEA both highlighted that monitoring remains insufficient across Member States, leading to the slow response in adapting regulatory frameworks and the need for a more standardized approach to data collection [54]. In connection with the above, the European Commission proposed expanding the possibilities of using new tools for obtaining monitoring data, including the Copernicus system or obtaining data obtained through citizen science. In addition, in order to make the flow of information more efficient and effective, the European Commission obliged the Member States to provide monitoring data to both the Commission and the EEA using automatic reporting mechanisms. The European Parliament, in its amendments, proposed the creation of a joint monitoring institution whose purpose would be to help the Member States develop new measurement methods and, at the same time, obliged the European Commission to analyze how such an institution could be created and function [54].

Another problem that was pointed out was the problem of the incomplete and outdated list of priority substances. The last update to the list occurred in 2013, and while updates are required at least once every four years (as per article 16(4) of the WFD), significant delays in publication have been noted. Although the list of priority substances has been expanded, including pharmaceuticals, this number is relatively small compared to international monitoring initiatives [55,56]. For example, a study conducted as part of the Global Monitoring of Pharmaceuticals project, which is one of the largest and most comprehensive monitoring programs of 61 pharmaceutical compounds in 104 countries, showed that pharmaceutical contaminants were detected in over 75% of the sampling sites, with some rivers showing contamination by as many as 34 different pharmaceuticals [56]. Despite these initiatives and still additions, the total number of pharmaceuticals under surveillance remains relatively low compared to the vast number of pharmaceutical substances potentially present in the environment. This discrepancy between known contaminants and those legally recognized as priority substances under the directive presents a significant regulatory lag.

Thus far, the time of introducing changes to the proposed Watch List has been extensive. This was due to the availability of scientific research data but primarily because of the lengthy legislative process. Therefore, it was proposed to transfer power to the European Commission to update the lists under Article 290 of the TFEU (delegated acts) [17] and not, as it has so far, under the ordinary legislative procedure based on the provisions of the WFD and GWD. Such a change should undoubtedly speed up the update of both the list of priority substances and the Watch Lists.

#### 3.3.3. Pharmaceutical Contamination and AMR Indicators

The inclusion of AMR indicators is a potential step towards more comprehensive water management in the context of counteracting pharmaceutical pollution, including antibiotics and reducing AMR. It should be noted that for the first time in water law there is a direct reference to AMR by including indicators of antimicrobial resistance evolution or transmission on the Watch List. Thus far, prevention of AMR has only been achieved by protecting waters from pharmaceutical contamination, including antibiotics. Monitoring pharmaceutical contaminants, including antibiotics, allows for the identification of the extent to which the environment is exposed to these compounds and thus can provide clues about the possibility of AMR. However, monitoring pharmaceutical contaminants is not enough to fully assess the threat associated with AMR. In order to obtain a more complete picture of the proliferation and transmission of AMR in water, it is necessary to introduce additional indicators, such as monitoring the presence of antibiotic-resistant bacteria and analyzing the transfer of resistance genes.

Monitoring both antibiotic-resistant bacteria and AMR genes provides complementary insights. Monitoring antibiotic-resistant bacteria offers a direct measure of the presence and potential impact of resistance on public health, as these bacteria are capable of causing infections. However, the presence of resistant bacteria can be transient and may not fully represent the broader environmental reservoir of resistance. In contrast, monitoring AMR genes provides a deeper understanding of the genetic potential for resistance within microbial communities, even without viable resistant bacteria. AMR gene monitoring is particularly valuable for tracking the mechanisms of resistance transmission across different environments, as these genes can be transferred between microorganisms through horizontal gene transfer.

While both approaches are essential, their utility depends on the monitoring objective. Monitoring antibiotic-resistant bacteria may be more relevant if the goal is to assess immediate risks to human health. Conversely, if the aim is to understand the potential for resistance spread and the long-term impact on ecosystems, monitoring AMR genes is a more powerful tool. Ideally, an integrated approach combining both methods would provide the most comprehensive picture of antimicrobial resistance dynamics in aquatic environments.

Although monitoring antibiotics in the environment alone is not a sufficient tool to assess the threat associated with AMR, together with the use of AMR indicators, such monitoring provides a more complete picture of the problem. This means that to effectively combat the problem of AMR, in the context of water pollution with pharmaceuticals, it should be based on both monitoring the pharmaceutical contaminants and indicators that are directly related to AMR.

Another problem is the lack of harmonized and reliable methods for monitoring AMR. The European Chemicals Agency (ECHA) will be required to prepare a scientific report containing the method of analysis and the maximum acceptable limit of quantification for each of the substances on the Watch List. Until now, each Watch List was introduced into the EU legal system by a commission implementing a decision, which, in addition to the list of compounds, including pharmaceuticals, included an indicative analytical method and maximum acceptable method of detection or quantification limit (see [33,34,35,36]).

#### 3.3.4. Innovative Monitoring Techniques

The new regulations also include the use of effect-based methods (EBMs) in monitoring, which reflects the increasing popularity of these methods in monitoring [57]. EBMs are monitoring techniques that focus on assessing the biological effects of chemical mixtures rather than measuring individual substance concentrations. They allow for evaluating the cumulative impact of contaminants on organisms and the environment, identifying potential toxic interactions and providing a more comprehensive risk assessment. The proposed methods can be used not only for the toxic effects of the pharmaceuticals determined for the endocrine system but also for other parameters, such as inhibition of respiration or the growth of organisms occurring in the environment [57,58,59]. These methods can determine the ecotoxicological effects of entire groups of substances found in water. Thanks to this, the effects of the interactions of compounds with each other are also observed. It also counteracts the risk associated with unknown chemical substances that are poorly researched and not subject to regulation. Currently, it is a useful tool in water management, complementing water quality assessments [60,61]. This approach excludes focusing on selected contaminants and reduces the monitoring costs by limiting cost-intensive chemical analytical method analyses and time-consuming analyses with the need to take into account low detection limits, which extends the sample preparation stage [57]. Unfortunately, the new regulations oblige Member States to apply EBMs in parallel with conventional chemical monitoring only for monitoring three estrogenic substances (17 alpha-ethinylestradiol (EE2), 17 beta-estradiol (E2) and Estrone (E1)) [51]. The European Commission was obliged to adopt guidelines for both chemical analysis methods and methods based on effects for estrogenic substances within 12 months of the new regulations coming into force. At the same time, in the WFD, in the section ‘Definitions’, point 35b was added, defining ‘Effect-based Trigger value’ as the effects of a pollutant or group of pollutants in water, sediment or biota, where those effects are measured by an appropriate and scientifically validated effect-based monitoring method, above which adverse effects on human health or the environment from that pollutant or group of pollutants in water, sediment or biota, could occur [51]. Effect-based trigger values were also included in the definition of ‘good surface water chemical status’, which opens the way to taking into account threshold values when determining the status of water, also for substances other than estrogenic substances.

#### 3.3.5. Monitoring Responsibility and Response to Data

Currently, the responsibility for monitoring water pollution lies predominantly with individual Member States [29], which may lead to significant disparities in the methods, the frequency of measurements and the data availability. Enhancing collaboration and coordination among EU Member States is essential to address these challenges and more effectively combat threats related to pharmaceutical contamination and AMR.

Each Member State is required to monitor the substances from the Watch List, including AMR indicators, for a period of 24 months, with the monitoring period starting within nine months of the establishment of the Watch List. This means that Member States will have to adapt their monitoring and water management programs to include these indicators. The Member State selects measurement points and the frequency of monitoring (no less than once a year), taking into account, among other things, the possibility of the occurrence of given substances, e.g., antibiotics. Therefore, monitoring pharmaceutical substances in water (including antibiotics) can identify areas where the concentrations of these substances are high, which may indicate that AMR may spread in these areas. Each Member State makes available the monitoring results together with information on the representativeness of the monitoring stations and monitoring strategies, and ECHA then reviews the results and assesses which substances should be further monitored and which should be removed from the Watch List [51].

The European Commission could establish a centralized coordination mechanism or a joint monitoring institution. This body would assist Member States in developing standardized methods, managing shared databases and ensuring consistent implementation across the Union. If monitoring data were to indicate elevated levels of AMR in specific areas, coordinated actions should be considered to assess and mitigate the associated risks. These actions might include:

- Implementing advanced treatment technologies in wastewater plants;
- Introducing stricter regulations for emissions from key sources, such as pharmaceutical industries, hospitals and agricultural practices;
- Raising public awareness through targeted educational campaigns;
- Enhancing early warning systems to identify and address emerging AMR threats rapidly.

Integrating the monitoring results with risk management strategies could enable a more dynamic adaptation of water protection policies. This would provide Member States with the tools to respond more effectively to potential threats, ensuring the sustainable management of water resources and supporting the broader goals of sustainable development.

Until now, WFD has used the ‘one-out, all-out’ principle, meaning that the final status of a water body is not determined by the average of all the indicators used for the assessment but only by the worst-rated parameter [28]. Therefore, if one element has a ’moderate’ status and the rest of the elements have ’good’, the whole water body is classified as ’moderate’ [62]. Critics of this principle pointed out that despite improvements in some parameters in the overall classification, this progress is invisible. In addition, problems with the reliability or accuracy of the assessment results were pointed out, which causes a greater risk of obtaining a lower class of water body status [62,63,64,65]. As a result, it may cause the need to implement actions (and incur financial outlays) where it is not necessary. Both Member States and, among others, The International Network of Basin Organizations in Europe (EUROPE-INBO) have postulated to change the regulations. In connection with the above, the European Commission has proposed introducing new indicators at the EU level that would show improvements in the status of water bodies, even in a situation where not all indicators indicate a ‘good status’ [51].

**Figure 1:**
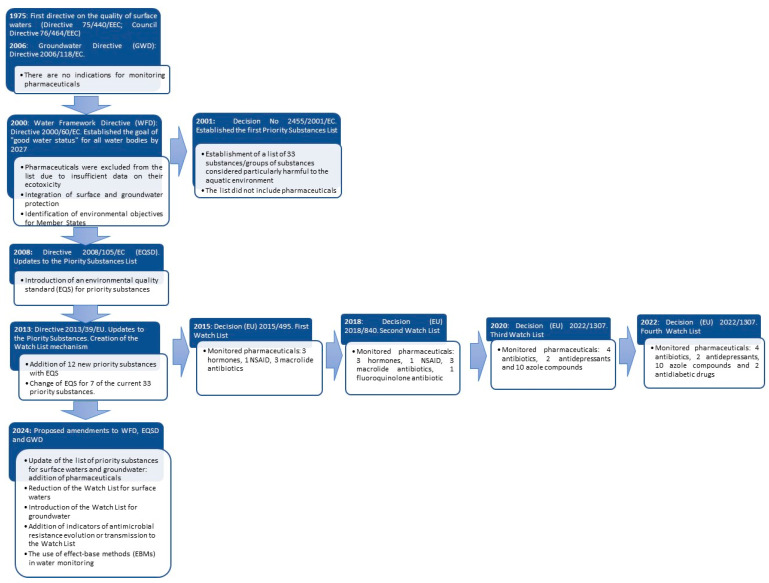
Changes to EU water law regarding pharmaceutical contaminants and AMR.

The evolution of EU water law regulations, along with the proposed changes in the context of preventing pharmaceutical and AMR pollution, is presented in Figure 1.

## 4. Urban Wastewaters—Nowadays and Future

The main source of pharmaceuticals, including antibiotics, entering the aquatic environment is sewage, both from the pharmaceutical sector and from households. It is estimated that about 50–100% of antibiotics in a given dose are eventually excreted in the urine and feces [66]. In order to protect the environment from the negative effects of the discharge of urban wastewater from agglomerations and industrial wastewater, in 1991, the Urban Wastewater Treatment Directive (UWWTD) was adopted [67]. Currently, the directive requires Member States to collect and treat municipal wastewater before it is discharged from agglomerations with a population equivalent (p.e.) of more than 2000. However, the requirements for municipal wastewater discharged from treatment plants only refer to parameters such as biochemical oxygen demand, chemical oxygen demand or total suspended solids [67]. In the case where the treated wastewater is to be discharged into waters defined as particularly sensitive and exposed to eutrophication, the directive provides the need to analyze the content of phosphorus and nitrogen and specifies their maximum permissible concentrations. In connection with the above, wastewater treatment plants operating in the EU are designed mainly to remove phosphorus, nitrogen or suspended solids [67]. First of all, mechanical–biological methods are used in these treatment plants. They are not able to effectively remove micropollutants such as antibiotics and antibiotic-resistant bacteria (ARB) from sewage, which causes them to enter the aquatic environment [68]. For example, research conducted by Kortesmäk et al. [69] in three Finnish wastewater treatment plants showed that while the efficiency of sulfonamides removal was between 58 and 100%, the concentration of macrolides was higher in the treated wastewater than in the influent. As shown by Kołecka et al. [70], some pharmaceuticals like ibuprofen, naproxen and diclofenac persist through treatment processes. Moreover, a correlation between substance sales data and their concentrations in wastewater was observed, finding that improper disposal likely contributes to elevated levels of sewage influents. Furthermore, the complex composition and high concentrations of pharmaceutical pollutants in wastewater —including residual drugs like carbamazepine and tetracyclines—reduce biodegradability, limiting the efficacy of traditional biological purification methods [71,72,73]. The increase in the efficiency of wastewater treatment is associated with the use of more advanced methods such as ozonation, adsorption onto zeolites or sorption onto activated carbon. That is why, in 2022, the European Commission proposed a revised UWWTD, not only to combat pollution from sewage more effectively but also to align it with the objectives of the European Green Deal. In January 2024, the Council and the Parliament reached a provisional agreement on the European Commission’s proposal. The most important changes to the directive include:

- Covering agglomerations above 1000 p.e. with the collected municipal sewage system by 31 December 2035;
- By 31 December 2039, all municipal sewage treatment plants with a load of at least 150,000 p.e. should have a tertiary treatment stage covering the removal of nitrogen and phosphorus;
- In the case of agglomerations of 150,000 p.e. and more (and over 10,000 p.e. based on a risk assessment), the fourth stage of sewage treatment from so-called micropollutants (including pharmaceuticals) must be introduced by 2045 unless the Member State proves that micropollutants do not pose a risk to human health and the environment. The directive indicates that the load of 10 pharmaceuticals in treated sewage should be reduced by 80%, including clarithromycin, carbamazepine, citalopram and metoprolol;
- Burdening, in accordance with the ‘polluter pays’ principle, producers introducing products containing substances that are micropollutants to the market with the costs (80%) associated with the introduction of the fourth stage of wastewater treatment. (including investment and operational costs). In connection with the fact that currently, the main micropollutants in wastewater are residues of cosmetic and pharmaceutical products, extended responsibility would apply primarily to the pharmaceutical and cosmetics industries. The Commission also envisages including other industrial sectors if the scientific research indicates that their activities may also be a source of micropollutants. The possibility of exemption from the obligations resulting from extended producer responsibility is to apply in situations in which the total content of substances in products is below 1 ton per year or when the producer proves that their product does not produce micropollutants or that the pollutants that do occur are biodegradable [74].

Extended producer responsibility in environmental protection is not a new mechanism and has been functioning for years as an economic policy instrument in the area of packaging waste or used electrical equipment. It is based on the assumption that producers are responsible throughout the product life cycle, including the post-consumer stage [75]. In some countries, such as France and Spain, this scheme is used to finance the collection and disposal of household pharmaceutical waste [76]. It seems that the introduction of extended producer responsibility in the area of wastewater treatment was only a matter of time, especially since the *Strategic Approach to Pharmaceuticals in the Environment* indicated that the fight against environmental pollution with pharmaceuticals should be based on the polluter pays principle, especially in relation to production processes [46]. The introduction of extended producer responsibility in the area of wastewater treatment was also recommended by the European Federation of National Associations of Water Services (EurEau). EurEau indicates that such a solution will prevent the transfer of the costs of wastewater treatment from micropollutants to consumers [77]

Problems that arise in the case of introducing the fourth stage of wastewater treatment from pharmaceuticals, including antibiotics and the burden of costs on producers, concern primarily economic and technological issues. Pistocchi et al. [78] indicated that reducing the total toxic load by about 75% as a result of modernizing existing wastewater treatment plants will generate a cost of about 4 billion euros per year for the EU. Some countries, despite the lack of European regulations, have already started to modernize their sewage treatment plants, e.g., Germany. It has been estimated that the cost of modernizing 230 German sewage treatment plants over a 25-year period may amount to 10.4 to almost 11 billion euros, or 415 to 435 million euros per year [79]. The directive indicates that the producer’s financial contribution should be proportional to the amount of substances contained in the products and the environmental hazard that these substances pose. This means that the contribution of different producers to pollution should be tracked and their relative size and hazard assessed, which may prove extremely difficult [80]. Moreover, the introduction of another purification step alone may not solve the problem of both antibiotics and ARBs in wastewater to the extent expected. On the one hand, Wang and Chen [81] indicate that ozonation can remove more than 99% of antibiotic-resistance genes. On the other hand, Sabri et al. [82], in their study conducted in three sewage treatment plants, showed that although good removal (79–88%) of antibiotics was observed in all sewage treatment plants, sulfonamides and quinolones were still present in the sewage in all three sewage treatment plants, regardless of whether it was a conventional treatment plant or one with additional technologies. Four-step wastewater treatment technology, while advanced and effective for many pharmaceutical compounds, has limited efficacy in completely removing some recalcitrant substances. Specific compounds, such as carbamazepine, fluoroquinolone antibiotics and some hormones, remain after treatment and are not removed by ozonation or granular activated carbon filtration. These techniques are insufficient to break down their chemical structures or adsorb them [83,84]. In addition, it should be noted that activated sludge can be a secondary source of both antibiotics and ARBs [82,85].

The advantage of introducing new regulations will undoubtedly be the search for new, safer-for-the-environment drugs by pharmaceutical companies. The emphasis on the production of more environmentally friendly drugs is also assumed by the new reform of EU pharmaceutical law. In 2023, the European Commission published a proposal for changes to the regulation and directive relating to medicinal products for human use, which were approved by the European Parliament on 10 April 2024. The proposals for changes both in the context of protecting the environment from pharmaceutical pollution and combating AMR concerns primarily are as follows:

- The obligation to conduct an environmental risk assessment of the medicinal product and to indicate the means of preventing this risk in the procedure of obtaining marketing authorizations. The risk assessment should cover both the production, use and disposal of the product. The lack, incomplete or insufficiently justified risk assessment will be the reason for refusing the authorization. In addition, the European Medicines Agency may oblige the entity already holding a marketing authorization for the medicinal product to conduct a post-approval environmental risk assessment if new evidence of its impact on the environment, including AMR, appears;
- Creation and maintenance by the European Commission of a register of environmental risk assessment studies for medicinal products;
- Expanding the concept of risk for using a medicinal product, which is related not only to the quality or safety of using this product by patients but also to the undesirable impact of this product on the natural environment or the negative impact related to AMR;
- Restricting the use of some antimicrobials by making them prescription-only;
- Introduction of special information on the packaging of antimicrobial products regarding their correct use and disposal as well as about AMR [86,87].

## 5. Conclusions

This article discusses the urgent challenges and strategies required to address water pollution by pharmaceuticals within the EU regulatory framework, with a focus on AMR and the emergence of new pharmaceutical contaminants in water. Pharmaceuticals, including antibiotics, pose a serious threat to aquatic ecosystems. Antimicrobial resistance is identified as a critical public health threat, and pharmaceuticals entering water systems contribute to the spread of resistant bacteria and genes. Legislative measures are increasingly focused on addressing water bodies as potential vectors of AMR, alongside the introduction of environmental risk assessments for pharmaceuticals. The evolution of the WFD and related directives shows an expanding scope to address pharmaceutical contaminants; however, existing provisions remain insufficient to address emerging contaminants. Although EU legislation has evolved to address this problem, the current measures face limitations. The European Commission’s strategic approach to pharmaceuticals in the environment, the updates of the Watch List and the inclusion of pharmaceuticals in the Watch List are important steps forward in addressing these challenges. However, legislative procedures need to be improved so that the substances identified as harmful can be added to the list of priority substances and regulated without delay. Failure to react quickly to new data can lead to further environmental degradation and health risks related to water-borne pollution. As the legislative proposals progress, the aim remains to improve the transparency, availability and efficiency of monitoring and data collection processes. The proposed changes aim to improve the standards for monitoring pharmaceuticals, although differences in monitoring capacities in Member States currently constitute a barrier to consistent water quality standards across the EU. The implementation of changes and proposed methods, sometimes associated with huge infrastructure costs, should be supported by appropriate EU programs. However, in parallel to monitoring chemicals, this should go hand in hand with the availability of ecotoxicological data for compounds. The problem is the limitations in monitoring all pharmaceutical products and new hazards, e.g., their transformation products. A solution could be the adoption of EBMs for monitoring, as they allow for a broader assessment of pollutant interactions and toxicological impacts on ecosystems without determining the concentration of all of them. Although the new legislative changes have included the use of EBMs, they do so only to a limited extent. Further research is also needed on pharmaceuticals and their potential contribution to AMR. The emphasis on ‘zero pollution’ targets underlines the need to integrate advanced treatment technologies to achieve sustainable development goals despite the significant investments required for implementation. However, this may not be sufficient to remove all types of pharmaceuticals in wastewater treatment plants, especially since the ‘polluter pays’ principle alone is insufficient for comprehensive protection. Therefore, emphasis should be placed on preventing the entry of micropollutants into the environment, especially into the water cycle. The persistence of micropollutants in water systems highlights the importance of preventive measures in addition to treatment solutions. In conclusion, EU water policy needs to become more flexible and responsive to new threats in order to better protect water resources and citizens’ health. Rapid implementation of scientific findings into water legislation is crucial to achieving the sustainable development and environmental protection goals, especially since the latest findings from the EEA 2024 report indicate that only 37% of EU surface waters achieved good or high ecological status in 2021, with little improvement since 2015—underlining the urgent need for more effective, adaptive strategies [88].

## Figures and Tables

**Table 1:** Pharmaceuticals on the Watch List.

Watch List (Year)	Compound	Type of Compound
**First Watch List (2015)**	17-alpha-Ethinylestradiol (EE2)	Hormone
	17-beta-Estradiol (E2)	Hormone
	Estrone (E1)	Hormone
	Diclofenac	Non-steroidal anti- inflammatory drug (NSAID)
	Erythromycin, Clarithromycin, Azithromycin	Macrolide antibiotics
**Second Watch List (2018)**	17-alpha-Ethinylestradiol (EE2)	Hormone
	17-beta-Estradiol (E2)	Hormone
	Estrone (E1)	Hormone
	Erythromycin, Clarithromycin, Azithromycin	Macrolide antibiotics
	Ciprofloxacin	Fluoroquinolone antibiotic
**Third Watch List (2020)**	Ciprofloxacin	Fluoroquinolone antibiotic
	Amoxicillin	β-lactam antibiotic
	Sulfamethoxazole	Sulfonamide antibiotic
	Trimethoprim	Antibiotic
	Venlafaxine and O-desmethylvenlafaxine	Non-tricyclic antidepressant
	Clotrimazole, Fluconazole, Imazalil, Ipconazole, Metconazole, Miconazole, Penconazole, Prochloraz, Tebuconazole, Tetraconazole	Azole compounds
**Fourth Watch List (2022)**	Sulfamethoxazole	Sulfonamide antibiotic
	Trimethoprim	Antibiotic
	Venlafaxine and O-desmethylvenlafaxine	Non-tricyclic antidepressant
	Clotrimazole, Fluconazole, Imazalil, Ipconazole, Metconazole, Miconazole, Penconazole, Prochloraz, Tebuconazole, Tetraconazole	Azole compounds
	Clindamycin	Antibiotic
	Ofloxacin	Antibiotic
	Metformin and Guanylurea	Antidiabetic drugs

**Table 2:** Watch List updates.

Watch List	Details
Expanded scope for groundwater monitoring	Inclusion of the requirement to develop a Watch List for groundwater (up to 5 substances/groups of substances).
Reduction in surface water Watch List	Reduction of the Watch List for surface water to 10 substances/groups of substances.
Update frequency	Updated every 3 years.
Extended monitoring period	Substances/groups of substances can remain on the Watch List for an additional 3 years if further monitoring is needed.
Inclusion of indicators of antimicrobial resistance evolution or transmission	Addition of indicators of antimicrobial resistance evolution or transmission to the Watch List once monitoring methods are available.

**Table 3:** Priority substance list updates.

Priority Substance List and EQS Updates for Surface Waters	Groundwater Quality Standards	National Threshold Values for Groundwater Contaminants
Increase the number of priority substances to 62, including pharmaceuticals: 17 alpha-ethinylestradiol (EE2), 17 beta-estradiol (E2), estrone (E1), azithromycin, erythromycin, clarithromycin, diclofenac, carbamazepine and ibuprofen.	Addition of 3 pharmaceuticals with groundwater quality standards: carbamazepine, sulfamethoxazole and primidone.	Establishment of threshold values for individual pharmaceutical active substances.

## Data Availability

No new data were created or analyzed in this study. Data sharing is not applicable to this article.
